# Genetic diversity of *Prunus armeniaca* L. var. *ansu* Maxim. germplasm revealed by simple sequence repeat (SSR) markers

**DOI:** 10.1371/journal.pone.0269424

**Published:** 2022-06-03

**Authors:** Jianhua Chen, Quangang Liu, Caiyun Lu, Qingbai Liu, Jingjing Pan, Jian Zhang, Shengjun Dong

**Affiliations:** College of Forestry, Shenyang Agricultural University, Shenyang, Liaoning, China; Institute of Mediterranean Forest Ecosystems of Athens, GREECE

## Abstract

The genetic diversity and genetic structure of *P*. *armeniaca* var. *ansu* were analyzed based on SSR markers. The aim was to provide scientific basis for conservation, efficient utilization, molecular marker assisted breeding and improved variety selection of *P*. *armeniaca* var. *ansu* germplasm resources. The results showed that the level of genetic diversity within the population was high. Among the 30 SSR markers, the mean number of observed alleles was 11.433, the mean number of effective alleles was 4.433, the mean of Shannon information index was 1.670, and the mean of polymorphic information content was 0.670. Among the eight provenances, Tuanjie Township, Xinyuan County, Xinjiang had the highest genetic diversity. The observed alleles, effective alleles, Shannon information index and Nei’s gene diversity index among provenances were higher than those within provenances. Based on Bayesian mathematical modeling and UPGMA cluster analysis, 86 *P*. *armeniaca* var. *ansu* accessions were divided into three subpopulations and four groups, which reflected individual differences in provenances. Subpopulations classified by Bayesian mathematical modeling and groups classified by UPGMA cluster analysis were significantly correlated with geographical provenance (Sig<0.01) and the provenances significantly impacted classification of groups. The provenances played an important role in classification of groups. The genetic distance between Tuanjie Township of Xinyuan County and Alemale Township of Xinyuan County was the smallest, while the genetic relationship between them was the closest and the degree of genetic differentiation was small.

## Introduction

*Prunus armeniaca* L. refers to both the wild progenitor and the cultivated species, which belongs to the family Rosaceae [1]. This species is an important stone fruit that is widely grown in the temperate regions of the world, and has an annual worldwide production of ~4.1 million tons (FAO, 2019). It is native to the Yellow River Basin in China, widely distributed across the mid-temperate zone and warm temperate zone of China, and covering most of the northeast, northwest, north and southwest regions [2]. It is also cultivated in the Mediterranean region, Middle East, Caucasus, and Central Asia [1].

*P*. *armeniaca* var. *ansu* is a sub-species of *Prunus armeniaca* L. [3]. This species is light-loving, drought resistant and cold resistant with its robust tree potential and strong adaptability [4]. It can be used as rootstock to graft peach and apricot of the same family, which can improve the cold resistance and stress resistance of these species. It is suitable for planting in barren slope areas with water shortage and thin soil layer. Therefore, it is one of the preferred tree species for returning farmland to forest and afforestation in barren mountains [5]. Its pollen is abundant and large, with a polar axis of about 59μm and an equatorial axis of about 28μm. The pollen lifespan is 40~50d when stored at ordinary temperature, and 90~105d when stored at 4°C. Therefore, it is suitable for pollination tree [6]. It has high nutritional and economic value and is utilized in various fields such as food, medicine, and industry [7]. It’s fruits with high soluble solids, edible fruit and kernels, and diverse colors and flavors, as well as later-blooming flowers, and late-maturing fruit [8]. The kernel oil can be used as high-quality raw materials for health-care vegetable oil, advanced lubricating oil, advanced cosmetics and advanced coatings [9]. The oil content reaches 53%, so *P*. *armeniaca* var. *ansu* is an excellent biodiesel species worthy of cultivation and popularization [10]. The kernel oil protein is a kind of plant protein with high edible value [9]. These excellent features are very valuable to the future genetic improvement and wider use of the species. *P*. *armeniaca* var. *ansu* is widely distributed in wild or semi-wild states [11], self-incompatibility and hybridization are common [12], and it has highly diverse germplasm. The diversity of *P*. *armeniaca* var. *ansu* germplasm enhances the breeding potential for this species, but also creates difficulties in identifying and classifying new accessions. Due to overgrazing and uncontrolled cutting, the area of the *P*. *armeniaca* var. *ansu* forest has been reduced greatly reduced, which threatens the existing biodiversity of this species. Moreover, with the increase of market demand, some excellent local varieties are disappearing, because they are replaced by more profitable species or a few improved varieties, so these rare local varieties are currently suffering serious genetic erosion. These genetic resources should be collected, conserved and used in breeding programmers so that they can be protected [13]. Information on genetic diversity and population structure is essential for developing management and conservation methods. Research aimed at understanding the genetic diversity of *P*. *armeniaca* var. *ansu* has important practical significance for the rational utilization and effective protection of these germplasm resources.

Previous analyses of the diversity of *P*. *armeniaca* var. *ansu* have primarily been based on phenotypic traits [4, 14], but the morphological indexes are susceptible to environmental influences. With the rapid development and improvement of molecular biology techniques, AFLP [15], RAPD [16], SRAP [17], ISSR [18] and SSR have all been widely deployed in germplasm evaluation [19], genetic map construction [15], genetic diversity assessment [20], genetic relationship analysis [13], and population structure analysis [21] in the genus *Prunus* Mill. Germplasm provides important resources for exploring and protecting the genetic and phenotypic diversity of breeding applications. Its genetic diversity determines the sustained ability of developing new high-quality varieties, which is essential to breeding sustainability and improvement [22]. Genetic diversity is the core of biodiversity, and is also the basis of collection, preservation, research, development, utilization and cultivar improvement of germplasm resources [23, 24]. SSR markers are extremely useful in such studies, due to their high polymorphism, co-dominant inheritance, great reproducibility, and ease of use [25]. Due to these benefits, SSR markers have been widely deployed in studying genetic diversity of species, including pear [26], taxus [27], jute [28], olive [29], and Jinsha pomelo [30].

*P*. *armeniaca* var. *ansu* germplasm resources are very abundant, and the study of their genetic diversity and genetic structure plays an important role in the protection, classification and utilization of this species. In this study, the genetic structure and diversity of 86 *P*. *armeniaca* var. *ansu* accessions were analyzed via SSR molecular markers in order to provide a scientific basis for conservation and efficient utilization of *P*. *armeniaca* var. *ansu* germplasm resources, as well as to provide guidance for the breeding of superior varieties.

## Materials and methods

### Plant materials

The plant materials consisted of 86 *P*. *armeniaca* var. *ansu* accessions, which were selected from 8 provenances in 2011. The detail geographic location information of 86 *P*. *armeniaca* var. *ansu* accessions was shown in S1 Table. These accessions were stored by asexual reproduction (grafting) in the National Forest Germplasm Resource Preservation Repository for *Prunus* species of Shenyang Agricultural University (Kazuo, Liaoning, China). Samples were collected in June 2020. The leaves of the one-year-old branches in the middle of the crown of the sample trees were collected. The collected leaves were first numbered and marked, wrapped in tinfoil, quickly frozen in liquid nitrogen, and stored at -80°C.

### DNA extraction

A genomic DNA extraction kit (Tiangen Biochemical Technology Co., Ltd., Beijing) was used to extract DNA. DNA quality was assessed via 1% agarose gel electrophoresis, and purity was tested by a NanoDrop 2000 spectrophotometer (NanoDrop, USA). The analyzed DNA samples were stored in a -20°C refrigerator until further use.

### SSR primer synthesis and PCR amplification

A total of 600 pairs of primers for *P*. *armeniaca* var. *ansu* were designed previously [31], 30 of which contained a high rate of polymorphism that were selected and synthesized by Beijing Saibaisheng Bioengineering Co., Ltd. (S2 Table). Amplification was carried out with a 20μl PCR reaction mixture of 20 ng template DNA, 0.125 μmol/L of each primer, 2.0 mmol/L Mg^2+^, 1.125 U Taq polymerase, and 0.45 mmol/L dNTPs. The PCR amplification reaction procedure was as follows: denaturation at 94°C for 5 min, followed by 34 amplification cycles (denaturation at 94°C for 30 s, annealing at 55°C for 30 s, with annealing temperatures adjusted according to the primers used in S2 Table, and extension at 72°C for 30 s), and a final extension at 72°C for 5 min. After the PCR amplification products were obtained, non-denatured polyacrylamide gel electrophoresis was performed, and after fixation, dyeing, rinsing and imaging, the products were photographed and recorded in the gel imaging system (BIO-RAD, USA).

### Statistical analysis

The gel image bands were analyzed by Image lab 4.0 software, and the data module of was used to determine uniform genotyping results. POPGENE version 1.32 was used to calculate the number of observed alleles (*N*_*A*_), the number of effective alleles (*N*_*E*_), observed heterozygosity (*H*_*O*_), expected heterozygosity (*H*_*E*_), percentage of polymorphic loci (PPL), Shannon’s information index (*I*), Nei’s gene diversity index (H), inbreeding coefficient (Fis), fixation index (Fit), genetic differentiation coefficient (Fst), gene flow (Nm), genetic distance and genetic similarity coefficient [32–34]. The Cervus version 3.0.7 was used to calculate polymorphic information content (*PIC*) [35]. The genetic similarity matrices between accessions were obtained using the SM similarity coefficient method in the NTSYS-pc 2.10e software. The clusters were then analyzed by the unweighted pair group method with arithmetic mean (UPGMA) to obtain a dendrogram [36]. STRUCTURE 2.3.4 was employed to analyze population structure based on a maximum likelihood mathematical model. The Bayesian clustering method in STRUCTURE was used to generate the genetic structure [37]. The calculations were carried out as described by [38], with default admixture and independent allele frequency models were utilized. K was set from 1 to 10, and each model run was repeated 10 times. The burn-in period was set to 100,000, followed by 100,000 MCMC iterations. The peak value of ΔK was used to determine the optimal K using STRUCTURE HARVESTER (http://taylor0.biology.ucla.edu/struct_harvest/) [39, 40]. Analysis of molecular variance (AMOVA) was performed using GenAlex6.502 [41]. Chi-square tests were conducted using SPSS 22.0 [42].

## Results

### Genetic diversity analysis of SSR markers in *P*. *armeniaca* var. *ansu*

The mean number of observed alleles from the 30 SSR markers was 11.433 and ranged from 3 to 23. The mean number of effective alleles was 4.433, and ranged from 1.151 to 12.016. The mean of Shannon information index was 1.670 and ranged from 0.285 to 2.773. The mean of the polymorphic information content was 0.670, and ranged from 0.125 (primer P3) to 0.912 (Table 1). The tested SSR markers revealed a high level of polymorphism and genetic diversity in *P*. *armeniaca* var. *ansu*. The mean values of PIC, as well as observed and expected heterozygosity, were found to be 0.670, 0.295 and 0.696, respectively. The expected heterozygosity of the 29 SSR markers was higher than the observed heterozygosity, accounting for 96.67% of all SSR markers (Table 1). These results indicated that the heterozygosity in *P*. *armeniaca* var. *ansu* population was low.

**Table 1:** Diversity of 30 SSR markers from *P*. *armeniaca* var. *ansu*.

Locus	Observed size range (bp)	Observed allele (*N*_*A*_)	Effective allele (*N*_*E*_)	Shannon’s information index (*I*)	Observed heterozygosity (*H*_*O*_)	Expected heterozygosity (*H*_*E*_)	Polymorphic information content (*PIC*)
**L23**	144~192	14	5.038	2.030	0.407	0.806	0.786
**L25**	112~136	8	3.981	1.635	0.256	0.753	0.718
**L46**	141~183	9	2.302	1.267	0.035	0.569	0.539
**L49**	132~156	9	4.388	1.785	0.198	0.777	0.748
**L62**	120~156	7	2.553	1.258	0.337	0.612	0.567
**L62H**	105~141	14	6.284	2.110	0.384	0.846	0.824
**L7**	114~132	7	3.014	1.441	0.174	0.672	0.638
**L70H**	135~174	13	3.588	1.728	0.407	0.726	0.693
**L75**	126~159	9	4.192	1.682	0.407	0.766	0.732
**L79H**	130~190	21	7.633	2.391	0.547	0.874	0.857
**P3**	123~129	3	1.151	0.285	0.070	0.132	0.125
**P21**	104~164	21	6.817	2.393	0.116	0.858	0.843
**P40H**	146~164	8	2.151	1.186	0.267	0.538	0.512
**P57H**	130~154	8	4.537	1.731	0.163	0.784	0.753
**X8H**	117~165	12	4.786	1.931	0.174	0.796	0.772
**X11H**	108~150	17	3.487	1.817	0.349	0.717	0.689
**X15H**	112~168	19	6.107	2.204	0.384	0.841	0.821
**X19H**	126~152	12	5.317	1.930	0.128	0.817	0.789
**X32H**	129~171	8	1.304	0.560	0.163	0.235	0.224
**X38H**	130~152	10	4.900	1.836	0.116	0.801	0.772
**X42H**	118~176	23	12.016	2.773	0.965	0.922	0.912
**X44H**	110~152	16	6.621	2.172	0.500	0.854	0.833
**X47**	135~160	6	1.795	0.890	0.337	0.445	0.412
**X58H**	120~162	15	8.681	2.384	0.174	0.890	0.875
**X70**	126~156	9	2.751	1.388	0.174	0.640	0.603
**X87**	120~144	9	4.294	1.662	0.523	0.772	0.733
**Y5**	128~164	8	2.114	1.188	0.140	0.530	0.503
**Y48**	117~147	10	3.129	1.590	0.221	0.684	0.657
**Y50**	144~153	5	1.593	0.741	0.279	0.374	0.347
**Y65**	134~166	13	6.457	2.097	0.454	0.850	0.828
**Mean**	/	11.433	4.433	1.670	0.295	0.696	0.670

### Genetic diversity of *P*. *armeniaca* var. *ansu* from different provenances

Among the eight provenances, the percentage of polymorphic loci ranged from 83.33% to 100%, with an average of 91.67%. Tuanjie Township and Qianjin Pasture had the highest percentages of polymorphic loci. The number of observed alleles ranged from 2.433 to 5.767, with an average of 3.746. Tuanjie Township had the highest number of observed alleles. The number of effective alleles ranged from 1.887 to 3.839, with an average of 2.710. The Shannon’s information index ranged from 0.646 to 1.400, with an average value of 0.988. Tuanjie Township had the highest Shannon’s information index. The observed heterozygosity ranged from 0.157 to 0.575, with an average of 0.365. Alemale Township had the highest observed heterozygosity. The expected heterozygosity ranged from 0.415 to 0.710, with an average of 0.590. Qianjin Pasture had the highest expected heterozygosity. The Nei’s gene diversity index ranged from 0.385 to 0.677, with an average value of 0.535. Tuanjie Township had the highest Nei’s gene diversity index (Table 2).

**Table 2:** Genetic diversity of *P*. *armeniaca* var. *Ansu* from different provenances.

Provenances	Sample size	Percentage of polymorphic loci % (PPL)	Observed allele (*N*_*A*_)	Effective allele (*N*_*E*_)	Shannon’s information index (*I*)	Observed heterozygosity (*H*_*O*_)	Expected heterozygosity (*H*_*E*_)	Nei’s gene diversity index
**Pengyang, Ningxia**	38	93.33	5.600	2.917	1.156	0.197	0.577	0.570
**Haiyuan, Ningxia**	7	83.33	2.433	1.887	0.646	0.157	0.415	0.385
**Zhenyuan, Gansu**	8	86.67	3.100	2.187	0.807	0.175	0.481	0.451
**Huining, Gansu**	7	93.33	2.833	2.273	0.839	0.162	0.534	0.496
**Tuanjie Township, Xinyuan County, Xinjiang**	15	100.00	5.767	3.839	1.400	0.536	0.701	0.677
**Qianjin Pasture, Xinyuan County, Xinjiang**	5	100.00	4.300	3.248	1.223	0.553	0.710	0.639
**Alemale Township, Xinyuan County, Xinjiang**	4	93.33	3.433	2.933	1.035	0.575	0.656	0.574
**Huocheng, Xinjiang**	2	83.33	2.500	2.393	0.796	0.567	0.650	0.488
**Mean**	10.75	91.67	3.746	2.710	0.988	0.365	0.590	0.535

### Molecular variance analysis within and among *P*. *armeniaca* var. *ansu* populations

AMOVA indicated that 83% of the genetic variation was found within *P*. *armeniaca* var. *ansu* populations, and only 17% of the variation occurred among *P*. *armeniaca* var. *ansu* populations (Table 3).

**Table 3:** Analysis of molecular variance (AMOVA) for *P*. *armeniaca* var. *ansu* populations.

Source of variation	Degree of freedom	Sum of squares	Mean of squares	Variance components	Percentage of variation
**Among populations**	7	421.479	60.211	2.294	17%***
**Within populations**	164	1817.730	22.824	11.412	83%
**Total**	171	2239.209		13.706	100%

*** significant data with *p* < 0.001.

### Analysis of the genetic structure of *P*. *armeniaca* var. *ansu*

**Fig 1:**
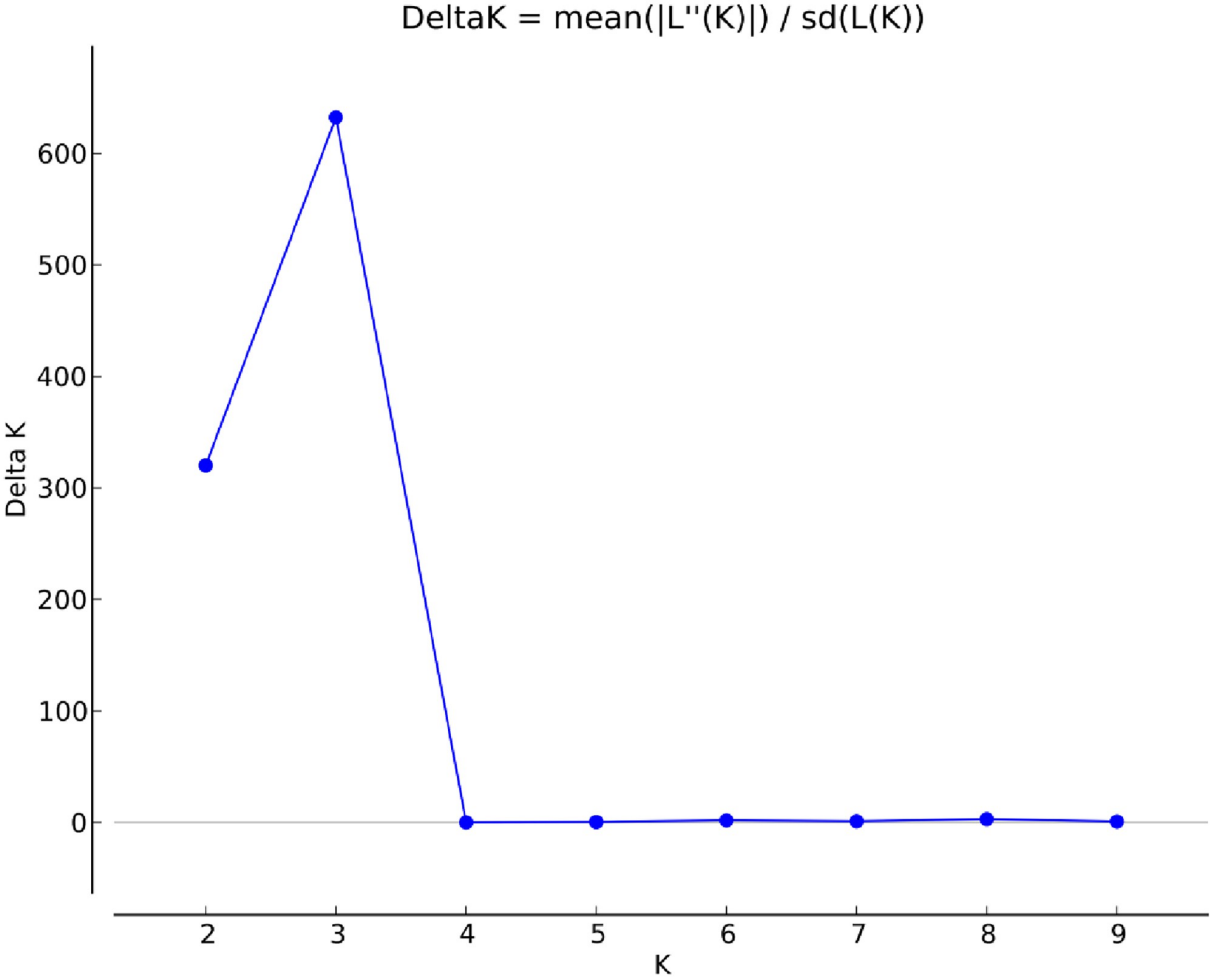
Determinations of subpopulations (K) of *P*. *armeniaca* var. *ansu* population.

Subpopulations were divided according to the ’Hierarchical Island’ model proposed by Evanno *et al*. (2005), in which the K value near the peak of ΔK was the closest to the actual number of subpopulations. When K was 3, ΔK was the largest (Fig 1). Therefore, the number of subpopulations of *P*. *armeniaca* var. *ansu* was determined to be 3 (K = 3). These results indicated that there were 3 subpopulations with different genetic structures.

**Fig 2:**
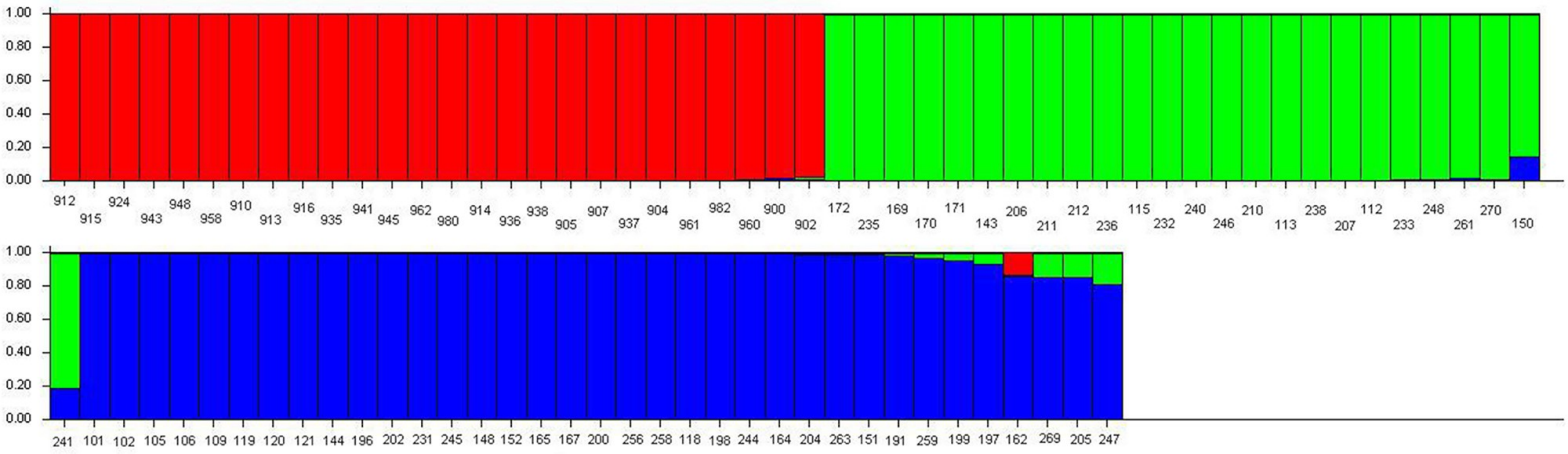
Q value distribution of *P*. *armeniaca* var. *ansu* K = 3. The 86 accessions were divided into subpopulations S1 to S3, comprised of 26 (red), 25 (green), and 35 (blue) accessions.

The drawing module of STRUCTURE 2.3 was used to create a bar graph of the Q value distribution under the optimal population structure (Fig 2). The Q matrix of *P*. *armeniaca* var. *ansu* (K = 3) was shown in S3 Table. *P*. *armeniaca* var. *ansu* accessions were divided into 3 subpopulations (S1, S2, and S3). The red bar graph in Fig 2 represented the first subpopulation (S1), which consists of 26 accessions from Xinjiang provenances, including Tuanjie Township (15), Qianjin Pasture (5), Alemale Township (4), and Huocheng (2). The green bar graph represented the second subpopulation (S2), which consists of 25 accessions from the northwest provenances, including Pengyang (3), Haiyuan (7), Zhenyuan (8), and Huining (7). The blue bar graph represented the third subpopulation (S3), including 35 *P*. *armeniaca* var. *ansu* accessions, and all of which were from Pengyang. Further analysis indicated that there was gene exchange among the three subpopulations. The genes of some accessions in the first subpopulation (S1) came from the second subpopulation (S2) and the third subpopulation (S3), while the genes of some accessions in the second subpopulation (S2) came from the third subpopulation (S3). Additionally, the genes of some accessions in the third subpopulation (S3) came from the first subpopulation (S1) and the second subpopulation (S2).

### Analysis of genetic relationships of *P*. *armeniaca* var. *ansu*

**Fig 3:**
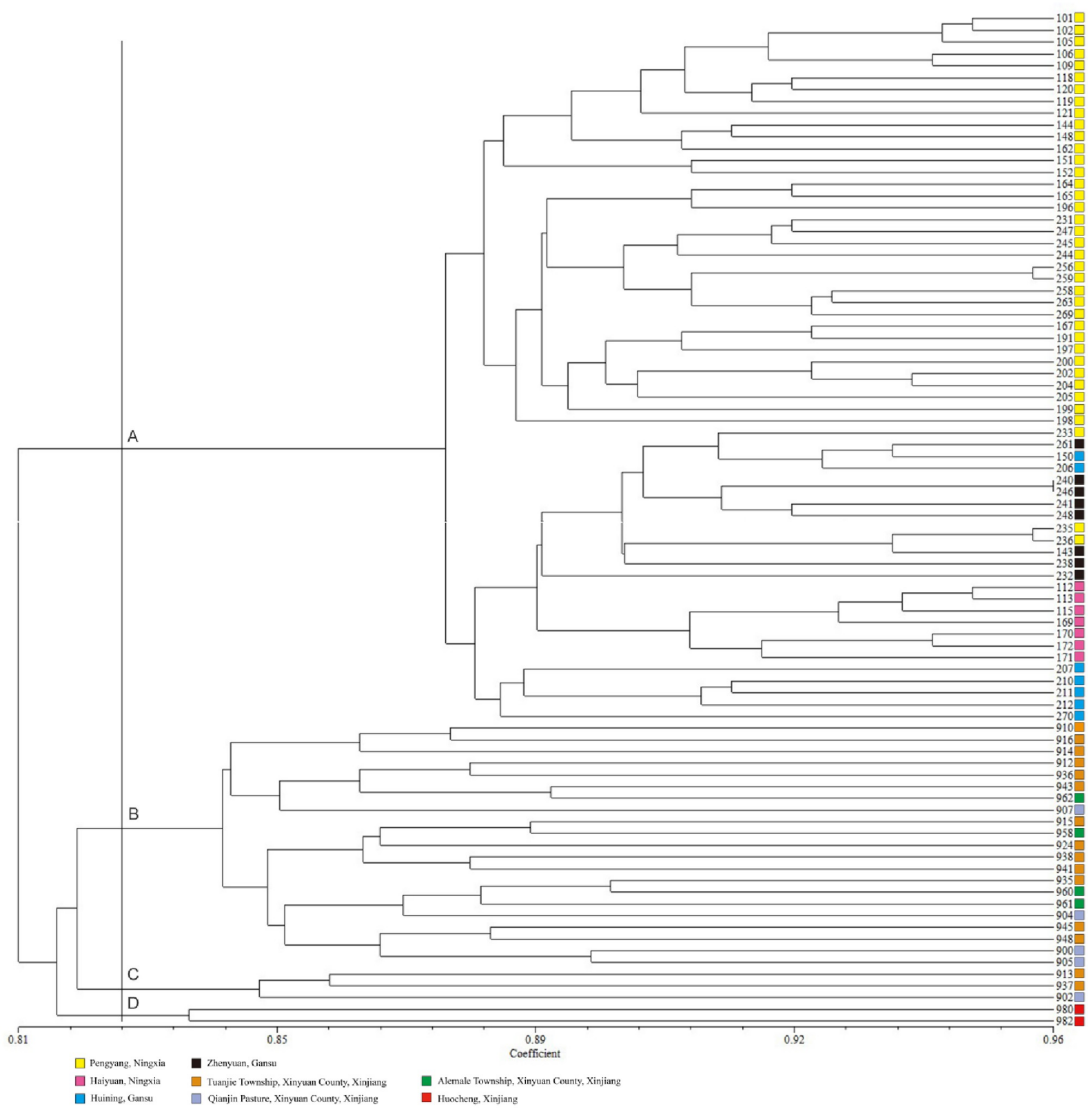
Dendrogram for UPGMA cluster analysis of *P*. *armeniaca* var. *ansu* based on SSR markers.

The UPGMA cluster analysis of 86 *P*. *armeniaca* var. *ansu* accessions based on SSR markers was shown in Fig 3. The genetic similarity coefficient ranged from 0.773 to 0.962. Entire accessions were could be divided into 4 groups (A, B, C, D in Fig 3), with a genetic similarity coefficient of 0.826. The accessions in Group A were all from Xinjiang provenances, while the accessions in Groups B, C and D were all from northwest provenances. The cluster analysis result reflected the differences in *P*. *armeniaca* var. *ansu* accessions across provenances. Group A included 60 accessions, which can be divided into 3 subgroups. The first subgroup included 35 accessions from Pengyang. The second subgroup was comprised of 20 accessions, including those from Zhenyuan (7), Haiyuan (7), Huining (3), and Pengyang (3). Group B contained 21 accessions, including those from Tuanjie Township (13), Alemale Township (4), and Qianjin Pasture (4). Group C included 3 accessions, which could be divided into 2 subgroups. The first subgroup included 2 accessions from Tuanjie Township, while the second subgroup included 1 accession from Qianjin Pasture. Group D included 2 accessions, both of which were from Huocheng.

### Correlations between subpopulations of *P*. *armeniaca* var. *ansu* based on mathematical modeling and groups based on UPGMA cluster analysis

Chi-square tests indicated that the correlations between 4 groups based on the UPGMA cluster analysis and 3 subpopulations based on mathematical modeling were highly significant (Sig<0.01) (Table 4).

**Table 4:** Correlations between subpopulations of *P*. *armeniaca* var. a*nsu* based on mathematical modeling and groups based on UPGMA cluster analysis.

Subpopulations based on mathematical model	Groups based on UPGMA cluster analysis					*χ* ^*2*^
	1	2	3	4	Total	
**Q1**	0	21	3	2	26	χ^2^ = 86.000
**Q2**	25	0	0	0	25	df = 6
**Q3**	35	0	0	0	35	Sig = 2.049E-16

### Genetic structure analysis of *P*. *armeniaca* var. *ansu* from different provenances

Among the different provenances, *P*. *armeniaca* var. *ansu* had an inbreeding coefficient (Fis) of 0.328, a fixation index (Fit) of 0.499, and a gene flow (Nm) of 0.731. The genetic differentiation coefficient (Fst) among provenances was 0.255. The genetic variation among provenances was 25.5%, and the genetic variation within provenances was 74.5% (Table 5). These results indicated that there was genetic differentiation within and among provenances. The genetic variation of *P*. *armeniaca* var. *ansu* occurred primarily, within provenances, with a small amount occurring between provenances.

**Table 5:** Genetic differentiation among provenances of *P*. *armeniaca* var. *ansu*.

Sample size	Inbreeding coefficient (Fis)	Fixation index (Fit)	Genetic differentiation coefficient (Fst)	Gene flow (Nm)
86	0.328	0.499	0.255	0.731

The Nei’s genetic distance and genetic identity among provenances were shown in Table 4. Among the 8 provenances, the genetic distance between two provenances ranged from 0.180 to 1.204, with an average of 0.627. The genetic distance between Tuanjie Township and Alemale Township was the smallest (0.180), indicating a small degree of genetic differentiation. The genetic distance between Haiyuan and Huocheng was the largest (1.204), indicating a large degree of genetic differentiation. The genetic identity between two provenances ranged from 0.300 to 0.836, with an average of 0.558. The genetic identity between Tuanjie Township and Alemale Township was the largest (0.836), while the genetic identity between Haiyuan and Huocheng was the smallest (0.300). The genetic identity indices were negatively correlated with genetic distance indices (Table 6).

**Table 6:** Nei’s genetic distance and genetic identity of *P*. *armeniaca* var. *ansu* between provenances.

	Pengyang, Ningxia	Haiyuan, Ningxia	Zhenyuan, Gansu	Huining, Gansu	Tuanjie Township, Xinyuan County, Xinjiang	Qianjin Pasture, Xinyuan County, Xinjiang	Alemale Township, Xinyuan County, Xinjiang	Huocheng, Xinjiang
**Pengyang, Ningxia**		0.783	0.833	0.727	0.466	0.504	0.496	0.378
**Haiyuan, Ningxia**	0.244		0.738	0.696	0.437	0.447	0.480	0.300
**Zhenyuan, Gansu**	0.182	0.304		0.750	0.458	0.468	0.490	0.347
**Huining, Gansu**	0.318	0.363	0.288		0.434	0.429	0.425	0.358
**Tuanjie Township, Xinyuan County, Xinjiang**	0.763	0.828	0.780	0.835		0.827	0.836	0.604
**Qianjin Pasture, Xinyuan County, Xinjiang**	0.684	0.806	0.760	0.847	0.191		0.769	0.577
**Alemale Township, Xinyuan County, Xinjiang**	0.700	0.735	0.714	0.856	0.180	0.263		0.557
**Huocheng, Xinjiang**	0.973	1.204	1.060	1.027	0.504	0.550	0.585	

## Discussion

Genetic diversity is the sum total of the genetic information carried by a species, which reflects the adaptability and evolutionary potential of populations in the environment [43]. Species with high genetic diversity can better adapt to environmental changes and are also more susceptible to environmental influences [44]. Studies focused on molecular-level genetic diversity typically employ Shannon’s information index, as well as several other metrics to measure the genetic diversity of germplasm [45, 46]. In our study, 30 SSR markers were used to analyze the genetic diversity of *P*. *armeniaca* var. *ansu*. Examination of these markers in the *P*. *armeniaca* var. *ansu* population revealed high levels of genetic diversity (He = 0.696). This level of diversity was higher than that of *Prunus mume* (He = 0.497) [47], and *Prunus brigantina* (He = 0.48) [48], but lower than that of *Prunus sibirica* (He = 0.774) [21] and *Prunus armeniaca* (He = 0.792) [13]. The level of genetic diversity may be related to molecular markers, samples, environmental conditions, and other factors. For example, *P*. *armeniaca* var. *ansu* showed a higher level of genetic diversity than *Prunus armeniaca* in Iran (He = 0.63) [49] and Tunisia (He = 0.56) [50], but a lower level of genetic diversity than *Prunus armeniaca* in Turkey (He = 0.72) [51], China (He = 0.774) [21], and Pakistan (He = 0.77) [52]. Plant growth is influenced by the genotype, environment and management factors. Asexual reproduction can maintain the excellent characteristics of the female parent. Different clones may show different phenotypes in the same environment, and the same clone may show different phenotypes in a different environment [53]. Among the 8 provenances examined in our study, Xinjiang province had the highest level of genetic diversity, with populations from Xinyuan County in Ili Kazakh Autonomous Prefecture showing the most diversity. This result was consistent with the conclusion that Ili was the center of origin for cultivated apricots [12].

Plant populations are not randomly distributed but are structured in space and time [54]. In this study, the *P*. *armeniaca* var. *ansu* population was divided into 3 subpopulations with a Bayesian model (Fig 1), which is mostly consistent with geographical distribution patterns among provenances. *P*. *armeniaca* var. *ansu* samples from provenances which were relatively close geographically were primarily found in the same subpopulation and had higher degrees of gene exchange (Fig 2). UPGMA clustering analysis of 86 *P*. *armeniaca* var. *ansu* accessions showed that those classified into the same subpopulation had close genetic relationships (Fig 3). Overall, these findings suggested that the population genetic variation in *P*. *armeniaca* var. *ansu* is significantly impacted by geographical distribution. To further elucidate the relationships among the populations, we analyzed the genetic distance and genetic identity between provenances. This analysis revealed that genetic identity of *P*. *armeniaca* var. *ansu* between provenances was negatively correlated with geographical distance, implying that *P*. *armeniaca* var. *ansu* may have undergone a pattern of isolation by dispersal limitation [55]. This phenomenon is generally consistent with isolation by distance (IBD) [56], which has also been reported in *P*. *armeniaca* [23].

Genetic structure results from the joint action of mutation, selection, migration, and drift [54], and causes changes in allele frequency that result in genetic differentiation [43]. Assessment of genetic differentiation revealed that the variation in *P*. *armeniaca* var. *ansu* mainly existed within populations (83%), which is similar to earlier results seen in *Prunus sibirica* [57], *P*. *armeniaca* [58] and most tree species [43]. Nevertheless, the genetic differentiation among *P*. *armeniaca* var. *ansu* geographic groups showed a high level of genetic differentiation (Fst = 0.255> 0.015) [59], which suggests a relationship between environment and genetic differentiation. The result of F-statistics indicated a degree of inbreeding in the mating system of *P*. *armeniaca* var. *ansu* (0<Fit<1) [43], and the heterozygosity of the population was low (Fis = 0.328). All samples in this study were from Central Asia, where apricot is generally self-sterile [60]. Therefore, we speculate that the low heterozygosity found in the *P*. *armeniaca* var. *ansu* was likely caused by mating among close relatives rather than self-pollination. Considering the low gene flow in the *P*. *armeniaca* var. *ansu* population (Nm = 0.731<1) [43], migration may had little effect on its genetic differentiation.

In the forest ecosystem, the extinction of a tree species can produce a chain reaction, which can lead to the extinction of some local appendage species [61]. Our results show that there was a degree of inbreeding in *P*. *armeniaca* var. *ansu*. With the intensification of inbreeding depression, forest productivity and population survivability will decline, which may lead to the extinction of this species, so it is necessary to preserve *P*. *armeniaca* var. *ansu* genetic resources. Considering the decentralized and wide distribution of *P*. *armeniaca* var. *ansu*, according to the existing genetic diversity and genetic structure of its populations, we propose a conservation strategy that combining *in situ* protection and *ex situ* protection. Measures that can be taken for *in situ* protection include establishing nature reserves, forest reserves, prohibiting grazing, controlling the utilization degree of wild resources, and encouraging vigorous promotion of *P*. *armeniaca* var. *ansu* resources in suitable areas. However, it is difficult to implement *in situ* protection for all species, so *ex-situ* protection should be given more attention. In addition, increasing the tending management of stands could also contribute to the protection of *P*. *armeniaca* var. *ansu* resources [43]. The main objective in genetic resource conservation programs should be to maintain the highest possible level of genetic variability [62]. We have established a National Forest Germplasm Resource Preservation Repository for *Prunus* species, which requires seeds and scions from each population for *ex-situ* protection. We will also collect germplasm resources in greater breadth and depth in the future. Based on the results of genetic diversity of *P*. *armeniaca* var. ansu from different provenances, the resources of Tuanjie Township, Xinyuan County, Xinjiang should be protected first.

## Conclusion

The *P*. *armeniaca* var. *ansu* population had a high level of genetic diversity, with those from Tuanjie Township and Xinyuan County being the most diverse. The level of genetic diversity among provenances was higher than diversity within provenances, and there was genetic differentiation within and among provenances. The genetic variation of *P*. *armeniaca* var. *ansu* mainly occurred within provenances, with a small degree present between them. The genetic relationship between Tuanjie Township, Xinyuan County and Alemale Township, Xinyuan County was the closest, and the degree of genetic differentiation was the smallest. Provenances played an important role in the classification of groups, while geographical distance was closely related to genetic difference. These results highlight the importance of accounting for provenances in future breeding efforts. Taken together, the results of our study provide a new scientific basis for conservation, efficient utilization and breeding of *P*. *armeniaca* var. *ansu* germplasm.

## Supporting information

S1 Table The geographic location information of 86 *P*. *armeniaca* var. *ansu* accessions. (DOCX)

S2 Table Information about the 30 primer pairs. (DOCX)

S3 Table The Q matrix of *P*. *armeniaca* var. *ansu* (K = 3). (DOCX)

## References

[1] Groppi A, Liu S, Cornille A, Decroocq S, Bui QT, Tricon D, et al. Population genomics of apricots unravels domestication history and adaptive events. Nature Communications. 2021; 12: 3956. doi: 10.1038/s41467-021-24283-6 34172741 PMC8233370

[2] Wang LB. Resource investigation and distribution pattern of three *Armeniaca* species. Forest resources management. 2011; 5: 65–70.

[3] Hagen L, Khadari B, Lambert P, Audergon JM. Genetic diversity in apricot revealed by AFLP markers: species and cultivar comparisons. Theoretical and Applied Genetics. 2002; 105: 298–305. doi: 10.1007/s00122-002-0910-8 12582532

[4] Cao Q, Liao K, Liu J, Xu GX, Sun Q, Si HZ. Study on the Daxigou wild apricot fruit phenotypic diversity in Huocheng County, Xinjiang. Xinjiang Agricultural Sciences. 2016; 53(5): 791–798.

[5] Liu LM, Liu P, Liang FL, Xu Z, Zhang Z. Primary Study on Floral Syndrome and Breeding System of *Armeniaca Vulgaris* Lam. 2010; 47(04): 750–755.

[6] Liu LM, Liu P, Liang FL, Xu Z. Study on pollen vitality and stigma receptivity of *Armeniaca vulgari*. Journal of South China Agricultural University. 2010; 31(04): 86–89.

[7] Ma FW, Dong SJ, Liu MG, Yang ZD, Wu YL. Quantitative classification of germplasm resources of *Armeniaca vulgaris* in loess hilly region of China. Nonwood Forest Research. 2013; 31(4): 98–103.

[8] Zaurov DE, Molnar TJ, Eisenman SW, Ford TM, Mavlyanova RF, Capik JM, et al. Genetic resources of apricots (*Prunus armeniaca* L.) in Central Asia. HortScience. 2013; 48(6): 681–691.

[9] Wang LB. Geographic distribution and botanical characters of 3 *Armeniaca* plant in China.Forest research. 2010; 23(03): 435–439.

[10] Wang LB. Assessment of three Armeniaca species seed oils for biodiesel traits. Transactions of the CSAE. 2011; 27(S1): 138–142.

[11] Yu DJ. Flora of China, vol 38. Science Publishing House, Beijing. 1986.

[12] He TM, Chen XS, Xu Z, Gao JS, Lin PJ, Liu W, et al. Using SSR markers to determine the population genetic structure of wild apricot (*Prunus armeniaca* L.) in the Ily Valley of West China. Genetic Resources and Crop Evolution. 2007; 54(3): 563–572. doi: 10.1007/s10722-006-0013-5

[13] Zhang QP, Liu DC, Liu S, Liu N, Wei X, Zhang AM, et al. Genetic diversity and relationships of common apricot (*Prunus armeniaca* L.) in China based on simple sequence repeat (SSR) markers. Genetic resources and crop evolution. 2014; 61(2): 357–368. doi: 10.1007/s10722-013-0039-4

[14] Liu J, Liao K, Liu H, Cao Q, Sun Q, Zhao SR. Phenotypic diversity of wild apricot germplasm resources in Xinjiang. Acta Botanica Boreali-Occidentalia Sinica. 2015; 35: 1021–1030. doi: 10.7606/j.issn.1000-4025.2015.05.1021

[15] Vilanova S, Romero C, Abbott AG, Llácer G, Badenes ML. An apricot (*Prunus armeniaca* L.) F2 progeny linkage map based on SSR and AFLP markers, mapping plum pox virus resistance and self-incompatibility traits. Theoretical and Applied Genetics. 2003; 107(2): 239–247. doi: 10.1007/s00122-003-1243-y 12845439

[16] Yilmaz KU, Paydas-Kargi S, Dogan Y, Kafkas S. Genetic diversity analysis based on ISSR, RAPD and SSR among Turkish Apricot Germplasms in Iran Caucasian eco-geographical group. Scientia Horticulturae. 2012; 138: 138–143. doi: 10.1016/j.scienta.2012.02.017

[17] Li M, Zhao Z, Miao XJ. Genetic diversity and relationships of apricot cultivars in North China revealed by ISSR and SRAP markers. Scientia Horticulturae. 2014; 173: 20–28. doi: 10.1016/j.scienta.2014.04.030

[18] Liu MP, Du HY, Zhu GP, Fu DL, Tana WY. Genetic diversity analysis of sweet kernel apricot in China based on SSR and ISSR markers. Genetics and Molecular Research. 2015; 14(3): 9722–9729. doi: 10.4238/2015.August.19.4 26345904

[19] Lamia K, Hedia B, Jean-Marc A, Neila TF. Comparative analysis of genetic diversity in Tunisian apricot germplasm using AFLP and SSR markers. Scientia Horticulturae. 2010; 127(1): 54–63. doi: 10.1016/j.scienta.2010.09.012

[20] Li M, Zhao Z, Miao X, Zhou JJ. Genetic Diversity and Population Structure of Siberian apricot (*Prunus sibirica* L.) in China. International Journal of Molecular Sciences. 2013; 15(1): 377–400. doi: 10.3390/ijms15010377 24384840 PMC3907815

[21] Wang Z, Kang M, Liu H, Gao J, Zhang ZD, Li YY, et al. High-Level Genetic Diversity and Complex Population Structure of Siberian Apricot (*Prunus sibirica* L.) in China as revealed by nuclear SSR markers. PLoS One. 2014; 9(2): e87381. doi: 10.1371/journal.pone.0087381 24516551 PMC3917850

[22] Zhang QP, Zhang DY, Yu K, Ji JJ, Liu N, Zhang YP, et al. Frequent germplasm exchanges drive the high genetic diversity of Chinese-cultivated common apricot germplasm. Horticulture Research. 2021; 8: 215. doi: 10.1038/s41438-021-00650-8 34593777 PMC8484454

[23] Bao WQ, Wuyun T N, Wang L, Zhao H, Du HY. Genetic diversity and population structure of wild Apricot in Xinjiang revealed by SSR markers. Acta Botanica Boreali-Occidentalia Sinica. 2016; 36(11): 1757–1763. doi: 10.7606/j.issn.1000-4025.2016.11.2182

[24] Hedrick PW. Recent developments in conservation genetics. Forest Ecology and Management. 2004; 197(1–3): 3–19. doi: 10.1016/j.foreco.2004.05.002

[25] Campoy JA, Martínez-Gómez P, Ruiz D, Rees J, Celton JM. Developing microsatellite multiplex and megaplex PCR systems for high-throughput characterization of breeding progenies and linkage maps spanning the apricot (*Prunus armeciaca* L.) genome. Plant molecular biology reporter. 2010; 28(4): 560–568. doi: 10.1007/s11105-010-0186-0

[26] Brini W, Mars M, Hormaza JI. Genetic diversity in local Tunisian pears (*Pyrus communis* L.) studied with SSR markers. Scientia horticulturae. 2008; 115(4): 337–341. doi: 10.1016/j.scienta.2007.10.012

[27] Zhang DQ, Zhou N. Genetic diversity and population structure of the endangered conifer *Taxus wallichiana* var. *mairei* (Taxaceae) revealed by Simple Sequence Repeat (SSR) markers. Biochemical Systematics and Ecology. 2013; 49: 107–114. doi: 10.1016/j.bse.2013.03.030

[28] Zhang LW, Cai RR, Yuan MH, Tao AF, Xu JT, Lin LH, et al. Genetic diversity and DNA fingerprinting in jute (*Corchorus spp*.) based on SSR markers. The Crop Journal. 2015; (05): 48–54. doi: 10.1016/j.cj.2015.05.005

[29] Aksehirli-Pakyurek M, Koubouris GC, Petrakis PV, Hepaksoy S, Metzidakis IT, Yalcinkaya E, et al. Cultivated and Wild Olives in Crete, Greece-Genetic Diversity and Relationships with Major Turkish Cultivars Revealed by SSR Markers. Plant Molecular Biology Reporter. 2017; 35(6): 575–585. doi: 10.1007/s11105-017-1046-y

[30] Liu ZL, Wan SL, Yan CP, Hu ZD, He XH, Zeng PZ, et al. Genetic Diversity of Jinsha Pomelo and Its Closely-related Germplasms Assessed by SSR Molecular Markers. Agricultural Biotechnology. 2017. 6(5): 15–22. CNKI:SUN:AGBT.0.2017-05-004

[31] Zhang X. Genetic diversity based on SSR and its association analysis with phenotypic traits in *Armeniaca sibirica* superior clones. Dissertation, Shenyang Agricultural University. 2016.

[32] Jiao Y, Jia HM, Li XW, Chai ML, Jia HJ, Chen Z, et al. Development of simple sequence repeat (SSR) markers from a genome survey of Chinese bayberry (*Myrica rubra*). BMC Genomics. 2012; 13: 201. doi: 10.1186/1471-2164-13-201 22621340 PMC3505174

[33] Chen Y, Peng Z, Wu C, Ma Z, Ding G, Cao G, et al. Genetic diversity and variation of Chinese fir from Fujian province and Taiwan, China, based on ISSR markers. PLoS One. 2017; 12(4): e0175571. doi: 10.1371/journal.pone.0175571 28406956 PMC5391013

[34] Yao YX, Shang XP, Yang J, Lin RZ, Huai WX, Zhao WX. Genetic Variation May Have Promoted the Successful Colonization of the Invasive Gall Midge, *Obolodiplosis robiniae*, in China. Frontiers in genetics. 2020; 11: 387. doi: 10.3389/fgene.2020.00387 32362914 PMC7180195

[35] Deng K, Liu W, Wang DH. Relatedness and spatial distance modulate intergroup interactions: experimental evidence from a social rodent. Current zoology. 2019; 65(5): 527–534. doi: 10.1093/cz/zoy082 31616483 PMC6784511

[36] Nie X, Tu J, Wang B, Zhou X, Lin Z. A BIL Population Derived from G. hirsutum and G. barbadense Provides a Resource for Cotton Genetics and Breeding. PLoS One. 2015; 10(10): e0141064. doi: 10.1371/journal.pone.0141064 26517274 PMC4627741

[37] Pritchard JK, Stephens M, Donnelly P. Inference of population structure using multilocus genotype data. Genetics. 2000; 155(2): 945–959. doi: 10.1093/genetics/155.2.945 10835412 PMC1461096

[38] Hubisz MJ, Falush D, Stephens M, Pritchard J K. Inferring weak population structure with the assistance of sample group information. Molecular Ecology Resources. 2009; 9(5): 1322–1332. doi: 10.1111/j.1755-0998.2009.02591.x 21564903 PMC3518025

[39] Evanno G, Regnaut S, Goudet J. Detecting the number of clusters of individuals using the software STRUCTURE: a simulation study. Molecular ecology. 2005; 14(8): 2611–2620. doi: 10.1111/j.1365-294X.2005.02553.x 15969739

[40] Earl DA, Vonholdt BM. Structure harvester: a website and program for visualizing STRUCTURE output and implementing the Evanno method. Conservation Genetics Resources. 2012; 4(2): 359–361. doi: 10.1007/s12686-011-9548-7

[41] Peakall R, Smouse PE. GenAlEx 6.5: genetic analysis in Excel. Population genetic software for teaching and research—an update. Bioinformatics. 2012; 28(19): 2537–2539. doi: 10.1093/bioinformatics/bts460 22820204 PMC3463245

[42] Lee J, Park KC, Sul HJ, Hong HJ, Kim KH, Kero J, et al. Loss of primary cilia promotes mitochondria-dependent apoptosis in thyroid cancer. Scientific reports. 2021; 11(1): 4181. doi: 10.1038/s41598-021-83418-3 33602982 PMC7893175

[43] White TL, Adams WT, Neale DB. Forest Genetics. Wallingford: CABI, 2007: 682.

[44] Yang M, Zhang M, Shi SG, Kang YX, Liu JJ. Analysis of genetic structure of *Magnolia sprengeri* populations based on ISSR markers. Scientia Silvae Sinicae. 2014; 50: 76–81.

[45] Wang HX. Analisis of genetic diversity and establishment of core Collection of Walnut varieties with AFLP markers. Dissertation, Hebei Agriculture University. 2006.

[46] Chen JH. Studies on mthod of constructing core germplasm collection and genetic diversity of *Boehmeria nivea*. Dissertation, Chinese Academy of Agricultural Sciences. 2011.

[47] Sw Kwon, Jw Chung, Jw Park, Lee GA, Ma KH, Lee MC, et al. Microsatellite variations and population structure in an on-farm collection of Japanese apricot (*Prunus mume* Sieb. et Zucc.). Biochemical systematics and ecology. 2012; 42: 99–112. doi: 10.1016/j.bse.2012.02.020

[48] Liu S, Decroocq S, Harte E, Tricon D, Chague A, Balakishiyeva G, et al. Genetic diversity and population structure analyses in the Alpine plum (*Prunus brigantina* Vill.) confirm its affiliation to the Armeniaca section. Tree genetics & genomes. 2021; 17(1): 2. doi: 10.1007/s11295-020-01484-6

[49] Raji R, Jannatizadeh A, Fattahi R, Esfahlani MA. Investigation of variability of apricot (*Prunus armeniaca* L.) using morphological traits and microsatellite markers. Scientia horticulturae. 2014; 176: 225–231. doi: 10.1016/j.scienta.2014.06.033

[50] Bourguiba H, Krichen L, Audergon J, Khadari B, Trifi-Farah N. Impact of Mapped SSR Markers on the Genetic Diversity of Apricot (*Prunus armeniaca* L.) in Tunisia. Plant molecular biology reporter. 2010; 28(4): 578–587. doi: 10.1007/s11105-010-0189-x

[51] Murathan ZT, Kafkas S, Asma BM, Topçu H. S_allele identification and genetic diversity analysis of apricot cultivars. The Journal of Horticultural Science and Biotechnology. 2017; 92(3): 251–260. doi: 10.1080/14620316.2016.1255568

[52] Ullah S, Muhammad A, Hussian I, Rahman HU, Hyder MZ. Geneticalysis of economically important apricot cultivars in gilgit baltistan based on SSR molecular markers. Romanian Biotechnological Letters. 2017; 22(2): 12456–12463. doi: 10.13140/RG.2.2.18610.15040

[53] Liang DY, Ding CJ, Zhao GH, Leng WW, Zhang M, Zhao XY, et al. Variation and selection analysis of Pinus koraiensis clones in northeast China. Journal of Forestry Research. 2018; 29: 611–622. doi: 10.1007/s11676-017-0471-y

[54] Loveless M D, Hamrick J L. Ecological Determinants of Genetic Structure in Plant Populations. Annual Review of Ecology and Systematics. 1984; 15(1): 65–95. doi: 10.1146/annurev.es.15.110184.000433

[55] Orsini L, Vanoverbeke J, Swillen I, Mergeay J, Meester LD. Drivers of population genetic differentiation in the wild: isolation by dispersal limitation, isolation by adaptation and isolation by colonization. Molecular Ecology. 2013; 22(24):5983–5999. doi: 10.1111/mec.12561 24128305

[56] Wright S. Isolation by Distance. Genetics. 1943; 28(2): 114–138. doi: 10.1093/genetics/28.2.114 17247074 PMC1209196

[57] Li M, Zhao Z, Miao XJ, Zhou JJ. Genetic diversity and population structure of Siberian apricot (*Prunus sibirica* L.) in China. International journal of molecular sciences. 2014; 15(1): 377–400. 10.3390/ijms15010377 PMC3907815 24384840

[58] Bourguiba H, Scotti I, Sauvage C, Zhebentyayeva T, Ledbetter C, Krška B. Genetic structure of a worldwide germplasm collection of *Prunus armeniaca* L. reveals three major diffusion routes for varieties coming From the Species’ Center of origin. Frontiers in plant science. 2020; 11: 638. doi: 10.3389/fpls.2020.00638 32523597 PMC7261834

[59] Balloux F, Lugon-Moulin N. The estimation of population differentiation with microsatellite markers. Molecular ecology. 2002; 11(2): 155–165. doi: 10.1046/j.0962-1083.2001.01436.x 11856418

[60] Paunovic SA. Apricot germplasm, breeding, selection, cultivar, rootstock and environmental. Acta Horticulturae. 1988; (209): 13–28. doi: 10.17660/actahortic.1988.209.1

[61] Ehrlich PR, Ehrlich A. Extinction. New York: Random House, 1981.

[62] Karp A. Molecular Tools in Plant Genetic Resources Conservation: A Guide to the Technologies. Rome, Italy: Bioversity International, 1997.

