# Supplementary material for: Genetic diversity of Prunus armeniaca L. var. ansu Maxim. germplasm revealed by simple sequence repeat (SSR) markers
Source: PLoS One. 2022 Jun 3;17(6):e0269424. doi: 10.1371/journal.pone.0269424 (PMC9165866; doi:10.1371/journal.pone.0269424)
Supplement: S1 Table — (DOCX) [file pone.0269424.s001.docx]

**S1 Table. The geographic location information of 86 *P. armeniaca* var. *ansu* accessions*.***

| **Accessions No.** | **Longitude** | **Latitude** | **Provenance** |
| --- | --- | --- | --- |
| **101** | 106.78416667 | 35.77777778 | Pengyang, Ningxia |
| **102** | 106.74805556 | 35.81694444 | Pengyang, Ningxia |
| **105** | 106.45666667 | 35.86333333 | Pengyang, Ningxia |
| **106** | 106.52416667 | 35.86416667 | Pengyang, Ningxia |
| **109** | 106.63722222 | 36.10861111 | Pengyang, Ningxia |
| **112** | 105.52833333 | 36.53750000 | Haiyuan, Ningxia |
| **113** | 105.67444444 | 36.54333333 | Haiyuan, Ningxia |
| **115** | 105.67194444 | 36.54138889 | Haiyuan, Ningxia |
| **118** | 106.52444444 | 35.86722222 | Pengyang, Ningxia |
| **119** | 106.80444444 | 35.79972222 | Pengyang, Ningxia |
| **120** | 106.71916667 | 35.88472222 | Pengyang, Ningxia |
| **121** | 106.67777778 | 35.92361111 | Pengyang, Ningxia |
| **143** | 107.06138889 | 35.64722222 | Zhenyuan, Gansu |
| **144** | 106.68000000 | 36.00777778 | Pengyang, Ningxia |
| **148** | 106.52444444 | 35.86638889 | Pengyang, Ningxia |
| **150** | 104.96963889 | 35.95211111 | Huining, Gansu |
| **151** | 106.65583333 | 36.09555556 | Pengyang, Ningxia |
| **152** | 106.64000000 | 36.10055556 | Pengyang, Ningxia |
| **162** | 106.73805556 | 35.89916667 | Pengyang, Ningxia |
| **164** | 106.79972222 | 35.79388889 | Pengyang, Ningxia |
| **165** | 106.65638889 | 35.77361111 | Pengyang, Ningxia |
| **167** | 106.06916667 | 35.93611111 | Pengyang, Ningxia |
| **169** | 105.52916667 | 36.53583333 | Haiyuan, Ningxia |
| **170** | 105.53000000 | 36.53611111 | Haiyuan, Ningxia |
| **171** | 105.52950000 | 36.53525000 | Haiyuan, Ningxia |
| **172** | 105.52916667 | 36.53500000 | Haiyuan, Ningxia |
| **191** | 106.65638889 | 36.09527778 | Pengyang, Ningxia |
| **196** | 106.65583333 | 36.09555556 | Pengyang, Ningxia |
| **197** | 106.65583333 | 36.09638889 | Pengyang, Ningxia |
| **198** | 106.65583333 | 36.09694444 | Pengyang, Ningxia |
| **199** | 106.65638889 | 36.09722222 | Pengyang, Ningxia |
| **200** | 106.65583333 | 36.09611111 | Pengyang, Ningxia |
| **202** | 106.65583333 | 36.09472222 | Pengyang, Ningxia |
| **204** | 106.65666667 | 36.09833333 | Pengyang, Ningxia |
| **205** | 106.65638889 | 37.09583333 | Pengyang, Ningxia |
| **206** | 104.96944444 | 35.95222222 | Huining, Gansu |
| **207** | 104.97027778 | 35.94944444 | Huining, Gansu |
| **210** | 104.97000000 | 35.95638889 | Huining, Gansu |
| **211** | 104.96944444 | 35.95222222 | Huining, Gansu |
| **212** | 104.97055556 | 35.94972222 | Huining, Gansu |
| **231** | 106.63277778 | 35.72916667 | Pengyang, Ningxia |
| **232** | 107.06166667 | 35.64722222 | Zhenyuan, Gansu |
| **233** | 106.49277778 | 35.84277778 | Pengyang, Ningxia |
| **235** | 106.77916667 | 35.80611111 | Pengyang, Ningxia |
| **236** | 106.49305556 | 35.84444444 | Pengyang, Ningxia |
| **238** | 107.22055556 | 35.79222222 | Zhenyuan, Gansu |
| **240** | 107.13305556 | 35.70722222 | Zhenyuan, Gansu |
| **241** | 107.21166667 | 35.79055556 | Zhenyuan, Gansu |
| **244** | 106.63277778 | 35.72944444 | Pengyang, Ningxia |
| **245** | 106.63305556 | 35.72972222 | Pengyang, Ningxia |
| **246** | 107.19611111 | 35.68277778 | Zhenyuan, Gansu |
| **247** | 106.73694444 | 35.82444444 | Pengyang, Ningxia |
| **248** | 107.20444444 | 35.72500000 | Zhenyuan, Gansu |
| **256** | 106.62472222 | 35.72250000 | Pengyang, Ningxia |
| **258** | 106.62388889 | 35.72166667 | Pengyang, Ningxia |
| **259** | 106.77750000 | 35.80583333 | Pengyang, Ningxia |
| **261** | 107.21166667 | 35.79055556 | Zhenyuan, Gansu |
| **263** | 106.67722222 | 35.92416667 | Pengyang, Ningxia |
| **269** | 106.74194444 | 35.82444444 | Pengyang, Ningxia |
| **270** | 104.98472222 | 35.81777778 | Huining, Gansu |
| **900** | 83.46938000 | 43.38789000 | Qianjin Pasture, Xinyuan County, Xinjiang |
| **902** | 83.46929194 | 43.38793389 | Qianjin Pasture, Xinyuan County, Xinjiang |
| **904** | 83.46925694 | 43.38792889 | Qianjin Pasture, Xinyuan County, Xinjiang |
| **905** | 83.46916306 | 43.38794306 | Qianjin Pasture, Xinyuan County, Xinjiang |
| **907** | 83.46884194 | 43.38796111 | Qianjin Pasture, Xinyuan County, Xinjiang |
| **910** | 83.49814306 | 43.37163389 | Tuanjie Township, Xinyuan County, Xinjiang |
| **912** | 83.49836000 | 43.37106000 | Tuanjie Township, Xinyuan County, Xinjiang |
| **913** | 83.49348056 | 43.37131944 | Tuanjie Township, Xinyuan County, Xinjiang |
| **914** | 83.49904444 | 43.37118611 | Tuanjie Township, Xinyuan County, Xinjiang |
| **915** | 83.49898611 | 43.37114444 | Tuanjie Township, Xinyuan County, Xinjiang |
| **916** | 83.49889444 | 43.37102500 | Tuanjie Township, Xinyuan County, Xinjiang |
| **924** | 83.50168889 | 43.37310556 | Tuanjie Township, Xinyuan County, Xinjiang |
| **935** | 83.49940833 | 43.37091111 | Tuanjie Township, Xinyuan County, Xinjiang |
| **936** | 83.49939194 | 43.37092889 | Tuanjie Township, Xinyuan County, Xinjiang |
| **937** | 83.49942306 | 43.37115500 | Tuanjie Township, Xinyuan County, Xinjiang |
| **938** | 83.49928611 | 43.37112889 | Tuanjie Township, Xinyuan County, Xinjiang |
| **941** | 83.49969694 | 43.37143500 | Tuanjie Township, Xinyuan County, Xinjiang |
| **943** | 83.49997222 | 43.37108389 | Tuanjie Township, Xinyuan County, Xinjiang |
| **945** | 83.49967194 | 43.37078306 | Tuanjie Township, Xinyuan County, Xinjiang |
| **948** | 83.49960694 | 43.37088389 | Tuanjie Township, Xinyuan County, Xinjiang |
| **958** | 83.58787000 | 43.38459000 | Alemale Township, Xinyuan County, Xinjiang |
| **960** | 83.58682194 | 43.38514694 | Alemale Township, Xinyuan County, Xinjiang |
| **961** | 83.58787500 | 43.38476500 | Alemale Township, Xinyuan County, Xinjiang |
| **962** | 83.58747000 | 43.38494000 | Alemale Township, Xinyuan County, Xinjiang |
| **980** | 80.98995833 | 44.37077500 | Huocheng, Xinjiang |
| **982** | 80.99009722 | 44.37072222 | Huocheng, Xinjiang |
