# Supplementary material for: Genetic diversity of Prunus armeniaca L. var. ansu Maxim. germplasm revealed by simple sequence repeat (SSR) markers
Source: PLoS One. 2022 Jun 3;17(6):e0269424. doi: 10.1371/journal.pone.0269424 (PMC9165866; doi:10.1371/journal.pone.0269424)
Supplement: S2 Table — (DOCX) [file pone.0269424.s002.docx]

**S2 Table.** **Information about the 30 primer pairs.**

| **Primer name** | **Repetition motif** | **Primer sequences (5'—3')** | **Temperature**  **(℃)** | **Length (bp)** |
| --- | --- | --- | --- | --- |
| **L23** | ATC | F: CAAATGTTGACATCTTGACGTGGT  R: TTGGTCTGTATTTGTGACGTGGTT | 63.278  62.739 | 160 |
| **L25** | ATGT | F: TACCAGCTAGCTATGACCCCAAAC  R: ACCGAAACAACCAGATTTGATCTC | 62.742  62.700 | 117 |
| **L46** | GTA | F: GCAGCCTTGAAAATCCCAAAA  R: GGCATGAGATGCACTATTTGACAC | 63.440  62.968 | 157 |
| **L49** | GTT | F: AGATCCTAGGCTGGAAGGCTCTAA  R: TCAGCCACTGCAAATAGCAATCTA | 62.995  63.184 | 158 |
| **L62** | TCCTCG | F: CTGGCAATGGCATTTATGTTGTAG  R: TTACCCTACCATCACCATGTAACG | 62.664  62.011 | 147 |
| **L62H** | TAT | F: TTCCTTGTCCTAAGCTTTGGTGTC  R: AATCAACCTCGACATGGAAAGTGT | 62.921  63.128 | 108 |
| **L7** | AAG | F: TTAGGGTACATGACACCAAGACGA  R: GCCAGGAATATCTTAGGGTTGGTT | 62.828  62.704 | 119 |
| **L70H** | TCT | F: CCACTCTCGTTTTACTTCCCAAGA  R: GGGGTGCATATAGATTTGAAGCAG | 62.905  63.034 | 144 |
| **L75** | TTC | F: GCTGTTTGCATTGGTCCATACTCT  R: CACTCAACTTATTCATCCAGACTCCA | 63.678  62.822 | 151 |
| **L79H** | TC | F: TAGGGATGAGCTGGCTGTTAGTTT  R: CCATGCCCAGGCCTATATAGAAGTA | 62.536  63.489 | 160 |
| **P21** | GA | F: GGGTTAGAGGTTCGTCGGAGTAGT  R: AAGCTCCACATTTCTTCATTGCTC | 63.105  63.039 | 112 |
| **P3** | ATG | F: AGGGCTTTCATTCCTTTAAGTTGG  R: GGGAGGAGACGAGTAGGGTAGAAA | 62.897  63.177 | 144 |
| **P40H** | GT | F: TTTGGTAAAAGACAACGACCCACT  R: TCCAACTCACACCCAAGTGATAGA | 63.028  63.026 | 155 |
| **P57H** | TC | F: CGCTATGGGGTAGGTTGTACATGA  R: CCCAAATATTTCAGGACCACAAGA | 64.160  63.300 | 140 |
| **X11H** | AG | F: TATATGTCAATGCTTGCACCCAAC  R: ATATCAACCTTGTAGCCCCCAACT | 63.076  63.102 | 123 |
| **X15H** | AG | F: TCCTTGCCTAGTCCAGCTTAAAAA  R: TGGCAGATGGAAACTCAAAGGTAT | 62.544  63.121 | 151 |
| **X19H** | AG | F: CGAACCTCTCCCTCTTTCTCTCTC  R: AAACCTAGCCCAAATCCTCCTTC | 63.273  62.972 | 149 |
| **X32H** | AGC | F: TACGCTTCAAACAAGTACAGCAGC  R: TGAGGCGAGAGAATAGATAAGAAGGA | 62.909  62.912 | 150 |
| **X38H** | AT | F: ATCCTAAAGGGCATCCCTCAAATA  R: TAGGGTTAAACGCCAAAATGTCAC | 63.075  63.240 | 151 |
| **X42H** | AT | F: GAAATACAATGCGACGTACACCCT  R: GGGGACATCATCTTGTAGTGCTG | 63.365  63.032 | 150 |
| **X44H** | AT | F: GAGGGATCTCTCAATAGAAGGGGA  R: TGGAGTACTCTCAATGCACATGCT | 63.148  63.296 | 122 |
| **X47** | CAGTC | F: ATCCGAATCCGATCGATTAAGTCT  R: CAAGTCCCTTCATGTTGTTCTGTG | 63.287  63.036 | 144 |
| **X58H** | CT | F: AGTGGGTAATGTGGCGAAGTAGAG  R: GTGTTTCTTCCATCTCCAGAAGCTA | 62.835  62.214 | 156 |
| **X70** | CT | F: AATTTGCAGAGACACCAAGGATG  R: GAGACTCTGACGACGGTTGAAGAT | 62.593  63.241 | 147 |
| **X87** | GGA | F: GGCCAGCCTCTTACTCAATAGACA  R: GTCGTCTAAACACAACACCCAACA | 63.035  63.285 | 124 |
| **X8H** | AAT | F: GTGTTGGTGTTTGGAGGTTTTCTC  R: GGGACATCCTTTAGGGTCCACTAC | 63.124  63.181 | 123 |
| **Y48** | CAC | F: CAGGATTCTGGAACAAGGAAGAGA  R: GAGAGGAGGTAGTGGGTGTCTTGA | 63.181  63.106 | 141 |
| **Y5** | AT | F: AAGGAGTGCAAGAATGAGTGAACC  R: GCAAGCCTTCTTCATATAGAGCCA | 63.027  63.046 | 148 |
| **Y50** | CAT | F: ATATCGCACACTGCAAACACTAGC  R: CGATTGCCATGGTCACTATTCTTA | 62.700  62.325 | 160 |
| **Y65** | GA | F: GAGAAGGAGACGAAGCTGTGAAAG  R: ACGAAATAGCGTCCAGATTCAATG | 63.023  63.547 | 159 |
