# Supplementary material for: Genetic diversity of Prunus armeniaca L. var. ansu Maxim. germplasm revealed by simple sequence repeat (SSR) markers
Source: PLoS One. 2022 Jun 3;17(6):e0269424. doi: 10.1371/journal.pone.0269424 (PMC9165866; doi:10.1371/journal.pone.0269424)
Supplement: S3 Table — (DOCX) [file pone.0269424.s003.docx]

**S3 Table.** **The Q matrix of *P. armeniaca* var. *ansu* (K=3).**

| **Accessions** | **Q1** | **Q2** | **Q3** | **Accessions** | **Q1** | **Q2** | **Q3** |
| --- | --- | --- | --- | --- | --- | --- | --- |
| **101** | 0.001 | 0.002 | 0.997 | **171** | 0.001 | 0.997 | 0.002 |
| **102** | 0.001 | 0.001 | 0.997 | **172** | 0.001 | 0.998 | 0.001 |
| **105** | 0.001 | 0.002 | 0.997 | **143** | 0.001 | 0.997 | 0.002 |
| **106** | 0.001 | 0.002 | 0.997 | **232** | 0.001 | 0.996 | 0.003 |
| **109** | 0.001 | 0.002 | 0.997 | **238** | 0.002 | 0.995 | 0.002 |
| **118** | 0.001 | 0.004 | 0.995 | **240** | 0.001 | 0.996 | 0.003 |
| **119** | 0.001 | 0.001 | 0.997 | **241** | 0.004 | 0.809 | 0.188 |
| **120** | 0.001 | 0.002 | 0.997 | **246** | 0.002 | 0.996 | 0.002 |
| **121** | 0.001 | 0.002 | 0.997 | **248** | 0.004 | 0.985 | 0.011 |
| **144** | 0.001 | 0.002 | 0.997 | **261** | 0.001 | 0.984 | 0.014 |
| **148** | 0.002 | 0.003 | 0.996 | **150** | 0.001 | 0.851 | 0.148 |
| **151** | 0.006 | 0.005 | 0.989 | **206** | 0.001 | 0.997 | 0.001 |
| **152** | 0.001 | 0.003 | 0.996 | **207** | 0.001 | 0.995 | 0.004 |
| **162** | 0.135 | 0.003 | 0.863 | **210** | 0.002 | 0.996 | 0.002 |
| **164** | 0.001 | 0.005 | 0.994 | **211** | 0.001 | 0.997 | 0.002 |
| **165** | 0.001 | 0.003 | 0.996 | **212** | 0.001 | 0.997 | 0.001 |
| **167** | 0.001 | 0.003 | 0.996 | **270** | 0.002 | 0.984 | 0.015 |
| **191** | 0.006 | 0.01 | 0.984 | **910** | 0.997 | 0.001 | 0.002 |
| **196** | 0.001 | 0.003 | 0.997 | **912** | 0.998 | 0.001 | 0.001 |
| **197** | 0.003 | 0.064 | 0.933 | **913** | 0.997 | 0.001 | 0.001 |
| **198** | 0.001 | 0.004 | 0.995 | **914** | 0.996 | 0.002 | 0.002 |
| **199** | 0.001 | 0.045 | 0.954 | **915** | 0.998 | 0.001 | 0.001 |
| **200** | 0.001 | 0.003 | 0.996 | **916** | 0.997 | 0.002 | 0.001 |
| **202** | 0.001 | 0.003 | 0.997 | **924** | 0.998 | 0.001 | 0.001 |
| **204** | 0.005 | 0.003 | 0.993 | **935** | 0.997 | 0.002 | 0.001 |
| **205** | 0.002 | 0.143 | 0.855 | **936** | 0.996 | 0.002 | 0.001 |
| **231** | 0.001 | 0.002 | 0.997 | **937** | 0.995 | 0.003 | 0.002 |
| **233** | 0.001 | 0.988 | 0.011 | **938** | 0.996 | 0.002 | 0.002 |
| **235** | 0.001 | 0.997 | 0.002 | **941** | 0.997 | 0.002 | 0.001 |
| **236** | 0.001 | 0.996 | 0.003 | **943** | 0.998 | 0.001 | 0.001 |
| **244** | 0.001 | 0.003 | 0.995 | **945** | 0.997 | 0.001 | 0.001 |
| **245** | 0.001 | 0.002 | 0.997 | **948** | 0.998 | 0.001 | 0.001 |
| **247** | 0.001 | 0.19 | 0.809 | **900** | 0.98 | 0.003 | 0.017 |
| **256** | 0.002 | 0.002 | 0.996 | **902** | 0.975 | 0.016 | 0.009 |
| **258** | 0.001 | 0.003 | 0.996 | **904** | 0.995 | 0.002 | 0.003 |
| **259** | 0.002 | 0.032 | 0.966 | **905** | 0.996 | 0.002 | 0.003 |
| **263** | 0.001 | 0.008 | 0.991 | **907** | 0.996 | 0.002 | 0.002 |
| **269** | 0.002 | 0.142 | 0.857 | **958** | 0.998 | 0.001 | 0.001 |
| **112** | 0.004 | 0.992 | 0.004 | **960** | 0.99 | 0.007 | 0.003 |
| **113** | 0.001 | 0.995 | 0.004 | **961** | 0.995 | 0.003 | 0.002 |
| **115** | 0.001 | 0.996 | 0.003 | **962** | 0.997 | 0.002 | 0.002 |
| **169** | 0.001 | 0.997 | 0.002 | **980** | 0.997 | 0.001 | 0.001 |
| **170** | 0.001 | 0.997 | 0.002 | **982** | 0.995 | 0.002 | 0.003 |
